# Effects of Particle Size on the Morphology and Water- and Thermo-Resistance of Washed Cottonseed Meal-Based Wood Adhesives

**DOI:** 10.3390/polym9120675

**Published:** 2017-12-05

**Authors:** Zhongqi He, Huai N. Cheng, K. Thomas Klasson, O. Modesto Olanya, Joseph Uknalis

**Affiliations:** 1 Southern Regional Research Center, USDA-ARS, 1100 Robert E Lee Blvd., New Orleans, LA 70124, USA; hn.cheng@ars.usda.gov (H.N.C.); thomas.klasson@ars.usda.gov (K.T.K.); 2 Eastern Regional Research Center, USDA-ARS, 600 East Mermaid Lane, Wyndmoor, PA 19038, USA; Modesto.Olanya@ars.usda.gov (O.M.O.); joseph.uknalis@ars.usda.gov (J.U.)

**Keywords:** biobased adhesive, cottonseed, Douglas fir, heat resistance, water resistance, white oak

## Abstract

Water washing of cottonseed meal is more cost-efficient and environmentally friendly than protein isolation by means of alkaline extraction and acidic precipitation. Thus, water-washed cottonseed meal (WCSM) is more promising as biobased wood adhesives. In this work, we examined the effects of the particle size on the morphology and adhesive performance of WCSM. Pilot-scale produced and dried WCSM was treated by three grinding methods: (1) ground by a hammer mill and passed through a 0.5-mm screen, (2) further ground by a cyclone mill and passed through a 0.5-mm screen, or (3) further ground by a ball mill and passed through a 0.18-mm screen. Micro-morphological examination revealed two types of particles. The filament-like particles were mainly fibrous materials from residual linters. Chunk-like particles were more like aggregates or accumulations of small particles, with proteins as the major component. Further grinding of the 0.5-mm Hammer product with the Cyclone and Ball mill led to more fine (smaller) particles in the WCSM products. The impact of further grinding on the dry and soaked adhesive strengths was minimal. However, the decrease of the hot and wet strengths of WCSM products by the additional grinding was significant (*p* ≤ 0.05). Data presented in this work is useful in developing the industrial standards of WCSM products used in wood bonding.

## 1. Introduction

Wood adhesives play a critical role in the wood products industry, as more than two thirds of wood products in the world today are totally, or at least partially, bonded together by adhesives or glues [1]. As synthetic wood adhesives are mainly derived from depleting petrochemical resources, prompting environmental concern, natural product- and byproduct-derived polymeric adhesives have attracted much more attention in the last decades [2]. Oilseed proteins, especially soy protein, are have been the most-studied for this purpose [3,4,5,6,7]. These proteins can be used directly, or further modified to improve their bond strength, operational ease, and/or cost [8,9,10,11]. 

Recently, our research group showed that cottonseed protein-based adhesives have great potential as sustainable eco-friendly adhesives [8,12]. Our data demonstrated that the bonding performance of cottonseed protein isolate was comparable to or even better than soy protein isolate under the same testing conditions [13,14,15]. In order to lower the cost and be more environmentally friendly in preparation, we further developed a new product, water-washed cottonseed meal (WCSM), as a promising biobased wood adhesive [16,17]. To promote practical application, WCSM was produced in a pilot scale [18], and the effects of several operational parameters on the bonding performance were evaluated [19,20,21].

Unlike protein isolate, WCSM contained both protein polymers and carbohydrate components [22]. The carbohydrates are composed of monosaccharide, cellulose, hemicelluloses and lignin [18,22], which could be either the indigenous portion of the cottonseed kernel or from the linter and hull residues [20,23]. As these polymeric carbohydrates are insoluble, it would be interesting to evaluate whether the particle size of WCSM products is able to affect WCSM’s bonding performance. Thus, in this work, we examined the morphology of three WCSM products, and tested their dry, wet, soaked, and heat shear strength on bonding white oak and Douglas fir veneers. The purpose of this work was to increase the morphological and performance knowledge of WCSM products for developing the industrial standards of WCSM as wood adhesives.

## 2. Materials and Methods

### 2.1. Materials

WCSM was prepared from mill-produced cottonseed meal on a pilot scale, with basic properties as described previously [18,20]. The pilot-produced WCSM was ground by a hammer mill (Model W-6-H, Schutte Buffalo Hammermill, Buffalo, NY, USA), passed through a 0.5-mm screen (WCSM-1). Part of WCSM was further ground by a cyclone sample mill (Model 3010-014, UDY Corporation, Fort Collins, CO, USA) and passed through a 0.5-mm screen (WCSM-2). Another part of WCSM was further ground by a ball Mixer/Mill (Model 8000M, SPEX Sample Prep LLC, Metuchen, NJ, USA) and passed through a 0.18-mm screen (WCSM-3). All the processed WCSM samples were kept in a desiccator at room temperature (22–23 °C) until use. White oak wood and Douglas fir veneers (1.59 mm thick) were purchased from Certainly Wood, Inc. (East Aurora, NY, USA). Veneers were cut in-house into strips 25.4 mm wide by 88.9 mm long, with the wood grain parallel to the long side, and were stored in sealed plastic bags at room temperature (22–23 °C) and room humidity (i.e., 50–60% relative humidity) until use. For wider validity of the results, these two types of wood were selected as they showed great differences in their physical properties [24]. The white oak was a hardwood with a hardness of 1360 lbf, a specific gravity of 0.68, and a modulus of elasticity of 1.78 million PSI. The Douglas fir was a softwood with a hardness of 710 lbf, a specific gravity of 0.48 and a modulus of elasticity of 1.95 million PSI. In addition, the surface of the Douglas fir was rougher than that of the oak, although with lower adhesive permeability [25,26]. 

### 2.2. Preparation of Bonded Wood Specimens

While most of the testing parameters were set as per the protocols for preparation and testing of plant seed meal-based wood adhesives reported previously, some parameters on post-bonding conditioning and treatments were adopted from European standards EN204 and EN14257 [27,28]. Adhesive slurries were prepared by mixing and stirring WCSM with appropriate deionized water to a solid content of 11% for 1 h at room temperature (22–23 °C). Using a brush, each slurry preparation was applied to one end of two wood veneer strips, covering 25.4 mm (1.0”) length [27]. After 10–15 min, a second layer was applied to the same strips. The tacky adhesive-coated areas of the wood veneer strips were overlapped and bonded by hot-pressing (Carver Benchtop Heated Press, Model 3856, Carver Inc., Wabash, IN, USA) at 2 MPa for 20 min at 130 °C. The bonded area between the two strips was 25.4 mm × 25.4 mm (1.0′′ × 1.0′′). These bonded wood specimens were cooled and set aside for conditioning at normal room temperature (22–23 °C) and relative humidity (50–60%) for 7 days before strength testing [28]. For each bonding condition, a total of 21 wood pairs were made. Five pairs were used for measuring the four types of adhesive strength described below. One pair was used for bondline examination by optical microscopy. Some of these bonding conditions were repeated once more so that the data of the strength measurements were presented with the average and standard deviation (*n* = 5 or 10). 

### 2.3. Adhesive Strength Testing

Further treatments of water soaking or heat exposure were conducted for those specimens used for water- and heat-resistance evaluation. Specifically, wet shear strengths were evaluated immediately after complete immersion of specimens in water for 4 days at 23 °C in reference to EN 204:2016 values [27]. Heat resistance was evaluated after exposing samples to the temperature of 80 °C for 1 h in an oven with well-circulated air, in accordance with EN14257:2006 [29]. The lap-shear strength of these preconditioned wood specimens was measured with a Zwick Materials Tester (Zwick GmbH & Co., Ulm, Germany). The bonded wood specimens were fitted into the 32 mm × 40 mm fishscale-gridded wedge grips on the Materials Tester with a gripping pressure of 7 MPa, and set the crosshead speed at 1 mm·min^−1^. The tensile shear strength at break for each bonded wood specimen was measured and recorded [26]. There was no observation of wood failure with these broken pairs. The shear strength at break (MPa) was reported as the adhesive strength of the tested WCSM products. 

### 2.4. Optical Microscopy

The WCSM products and bonded joints of the wood veneer strips were observed under a stereo microscope (Olympus SZX12, Tokyo, Japan) with 40× and 90× magnifications, respectively. The images were captured with a CCD camera (Olympus DP70, Tokyo, Japan). Prior to the analysis, cross sections of the wood substrates were prepared by razor-blade cutting. The wood substrates were stained with 0.01% aqueous safranin-O (Sigma Aldrich, St. Louis, MO, USA).

### 2.5. Scanning Electron Microscopy (SEM) and Scanning Electron Microscopy-Energy Dispersive Spectroscopy (SEM-EDS)

For SEM analysis, WCSM powders were mounted on aluminum stubs with double-sided carbon tape and sputter-coated with gold for 1 min (EMS 150R ES, EM Sciences, Hatfield, PA, USA) at 20 milli Amps. Samples were then viewed with a FEI Quanta 200 F Scanning Electron Microscope (Hillsboro, OR, USA) with an accelerating voltage of 10 KV in high-vacuum mode. The images of the samples were then scanned at 500× and 1000× (magnifications) for comparison of the treatment effects. 

For EDS processing, the same samples utilized above were mounted on aluminum stubs with double-sided carbon tape. Elemental analysis was done with an Oxford Xmax^n^ 80 mm^2^ Detector (Oxford Instruments, Tubney Woods Abingdon, Oxfordshire OX13 5QX, Hillsboro, OR, USA). Spectrum acquisition and interpretation was performed with AZtec software version 3.1 (Oxford Instruments, Hillsboro, OR, USA). Spectra were acquired at 30 KV and spot size of 5 (about 8.2 μm) for each sample.

## 3. Results and Discussion

### 3.1. Morphology of WCSM Prodcuts

**Figure 1:**
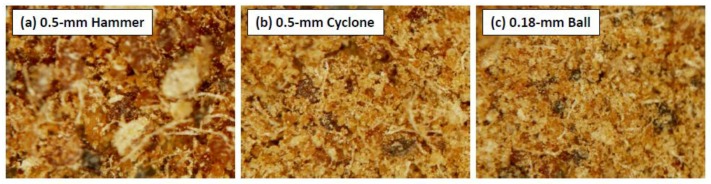
Stereomicroscopic images of WCSM products ground by three mills and passed by 0.5-mm and 0.18-mm screens, respectively.

The images of the WCSM samples ground by three mills are shown in Figure 1. The image of the WCSM sample passed through the 0.5-mm screen of the hammer mill showed some large bulks and long filaments. These fibrous filaments and bulky particles could be attributed to the residual linters and hulls of cottonseed. Indeed, some of these materials (8%) were >0.5 mm in particle size, and were forced by the hammer to pass through the 0.5-mm screen [20]. The presence of these residues was also supported by the chemical analysis [18], which reported that the pilot-produced WCSM contained 46.3% of protein, 16% of crude fiber, 9.4% of acid detergent lignin, 17.6% of cellulose, and 8.4% of hemicellulose. Further grinding by the cyclone mill apparently reduced the fraction of long filaments and large bulks. There were no remarkable differences in the morphology and homogeneity of the WCSM samples ground by the 0.5-mm cyclone mill and the 0.18-mm ball mill, although the latter looked finer.

**Figure 2:**
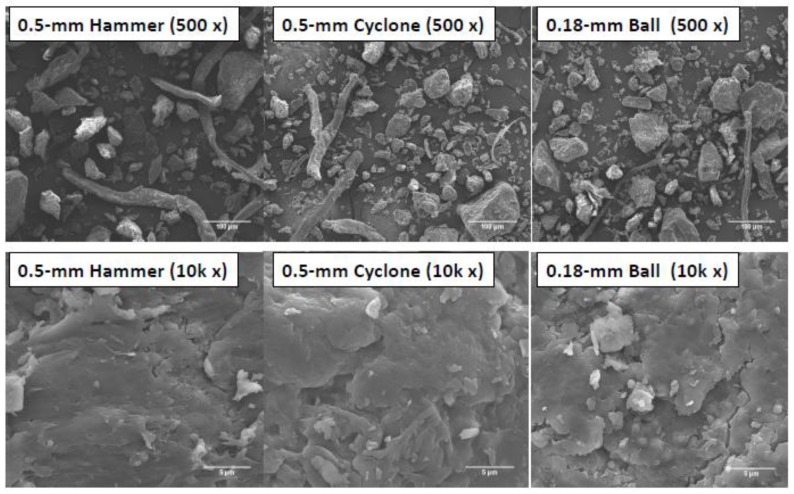
SEM images, with 500- and 10,000-times magnifications, of WCSM products ground by three mills and passed through 0.5-mm and 0.18-mm screens, respectively.

The WCSM particles were also examined by SEM (Figure 2). At 500× magnification, the SEM images showed both amorphous chunks (irregular shapes and sizes) and filament-shaped particles. These features are similar to, but clearer than, the images observed with optical microscopy (Figure 1). The filaments were mainly the fibrous materials from residual linters, and the chunk particles were proteins and other components (such as lignin). The images also showed that there were more fine particles in the two samples subjected to further grinding than the 0.5-mm Hammer mill-produced WCSM product. However, there was not much difference between the 0.5-mm Cyclone and the 0.18-mm Ball products. After all, it is logical that additional grinding of the 0.5-mm Hammer product would make the product smaller, regardless of whether it was passed through the same 0.5-mm size screen or a smaller 0.18-mm screen. The chunk particles were further examined at a higher magnification (i.e., 10,000×). These images (Figure 2) show that the chunk particles were more like aggregates or accumulations. While their surface was characterized by light smooth structures with flat areas and cracks, the fracturing and overlapping features increased with additional grinding by cyclone and ball mills. This observation might be an indicator of more protein being denatured in the additional grinding processes.

### 3.2. Surface Compostion of WCSM Products 

**Figure 3:**
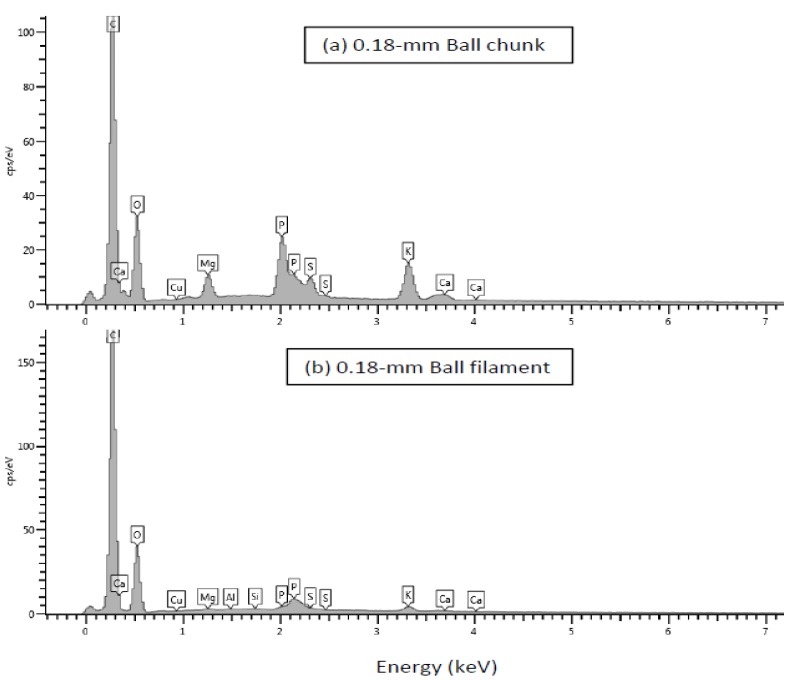
EDS spectrograms of the chunk and filament particles of WCSM products ground by the ball mill and passed by a 0.18-mm screen.

The surface composition of the chunk and filament particles of the three WCSM products was further analyzed by SEM-EDS. The representative spectrograms of WCSM products are presented in Figure 3. Strong peaks of C and O were detected at 0.29 and 0.53 KeV, as they were the major elements in organic materials. Several trace mineral elements (i.e., Ca, Cu, S, Mg, K and P) were also detected on the surface of these products. Although N was also a major component in the bulk WCSM samples, the N peak was not observed at the assumed 0.40 KeV in the EDS spectrograms of these WCSM products, while the N content in the three bulk samples was 7.40%, 6.70% and 7.59%, in order, obtained by chemical analysis. Thus, we ran a control EDS analysis of tryptophan (one of the 20 protein amino acids) and urea (a simple organic N compound). The N peak appeared at 0.39–0.40 KeV between the C and O peaks in the spectrogram of urea, but not in that of tryptophan (data not shown). While the N peak was observed in EDS analysis of industrial and environmental samples [30,31], the lack of N peaks has also been reported before, especially with biological samples [32,33]. This no-N-peak observation might be an indicator of a difference in the distribution of the N compound (i.e., protein, in these samples) between the surface and the bulk, as the electron beam of EDS with an accelerating voltage of 10 KV penetrates to a depth of about 200 nm [34], and the sample matrix may have significant effects on the detection of N by SEM-EDS [30]. The absence of N detection in these WCSM samples based on SEM-EDS analysis might suggest that the protein was inside the powder matrix, covered by a layer of polysaccharides and/or other components [33]. It is hypothesized that non-N atoms or groups of atoms in the protein itself could have shielded N from the surface of the protein structures, leading to its lack of detection. Further rigorous studies are needed to validate these assumptions as it could not be excluded that the very weak but broad wimple around 0.35–0.39 KeV in the spectrogram of the chunk particle sample could be co-contributed by Ca and N.

Quantitative data of the elements on the surface of the chunk and filament particles of the three WCSM products are listed in Table 1. Carbon accounted for about 60% of the dry matter, and O accounted for 30 to 43% of the dry matter. The minerals accounted for about 2% each or less of the dry matter. The C content of the filament-like particles was always higher than in the corresponding chunk-like particles in all three WCSM samples. Overall, the total mineral content (K, Mg, Ca, S, P and Cu) was lower in the filament-like particles than in the chunk-like particles for the same WCSM sample. Those mineral components could be either part of the proteins (e.g., metalloproteins, di-sulfide bonds) or small molecules associated with proteins or other biomolecules (e.g., phytate). These observations confirmed that the filament-like particles were carbohydrate-related in nature (i.e., fibrous components). There were more complicated components in the chunk-like particles. There was no consistent observation of O content among the three WCSM samples, as carbohydrates, proteins, and other components all contained O, but with varying stoichiometry. Compared to the chunk-like particles in the 0.5-mm Hammer samples, the contents of Mg, Ca and S increased in the chunk-like particles of the 0.5-mm Cyclone and 0.18-mm Ball samples. The P content was greatly increased in the 0.18-mm Ball sample. In other words, these elements were enriched on the surfaces of the WCSM samples after the additional grinding. These results might be due to the finer particle size or the heat denaturing effect with additional grinding, both of which could lead to higher exposure of the inter-surface structures of proteins and other associated components.

### 3.3. Effects of Particle Size of WCSM on the Shear Strength of White Oak and Douglas Fir Bonding Joints

**Figure 4:**
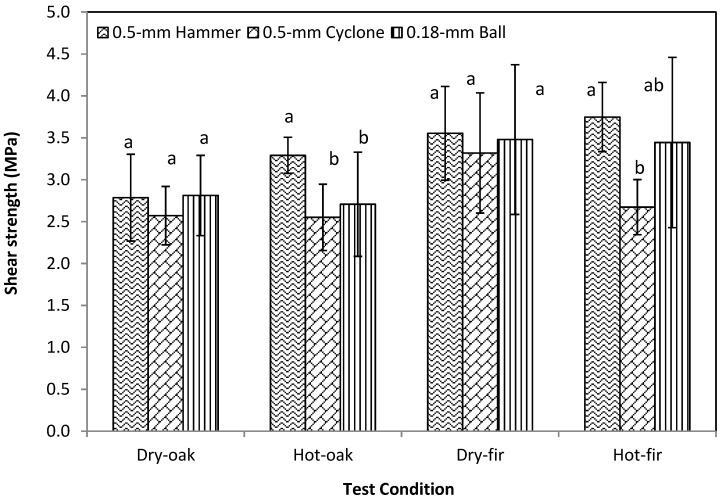
The dry and hot shear strength of white oak and Douglas fir veneer pairs bonded by three WCSM products. Data are presented as average ± SD (*n* = 10 but 5 for Dry-fir samples). Different letters in the same type of strength indicate the statistically significant difference between the values at *p* ≤ 0.05.

The pilot-produced WCSM showed a dry bonding strength of 2.79 MPa on oak strips, and 3.55 MPa on fir strips (Figure 4). Heat exposure of the bonded strips at 80 °C for 1 h increased the shear strength of the oak and fir specimens bonded with 0.5-mm hammer-ground WCSM to 3.29 and 3.75 MPa, respectively. Heat exposure did not show a remarkable impact on the bonding strength of WCSM with the other two mills. Indeed, the hot shear strength of the fir strips bonded by 0.5-mm cyclone was lower than the dry shear strength of the fir strips bonded by the same WCSM product. Little or negative impact of heat exposure on the adhesive strength of WCSM has also been observed previously with beech thin board [21]. 

### 3.4. Effects of Particle Size of WCSM on the Water Resistance of White Oak and Douglas Fir Bonding Joints

**Figure 5:**
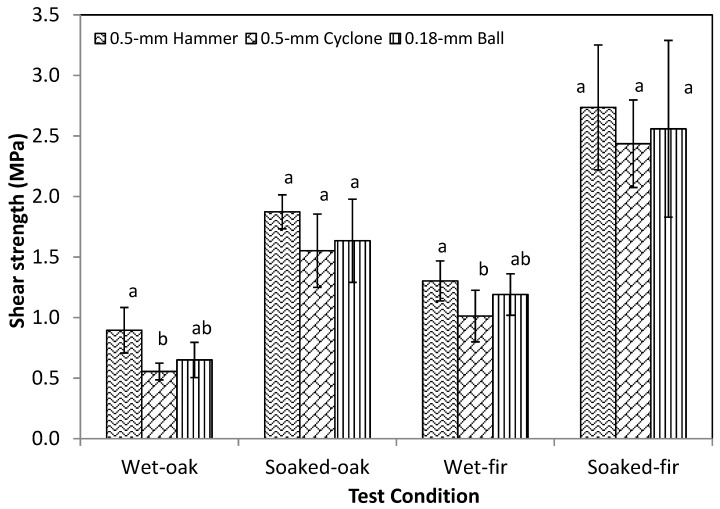
The shear strength of wet and soaked white oak and Douglas fir veneer pairs bonded by three WCSM products. Data are presented as average ± SD (*n* = 10 for oak samples and 5 for fir samples). Different letters in the same type of strength indicate the statistically significant difference between the values at *p* ≤ 0.05.

After water soaking, the shear strength of the bonding joint of all oak pairs was <1 MPa (Figure 5). The wet shear strength of the bonding joint of the fir pairs was higher, with values between 1.0 and 1.3 MPa, apparently due to their having a higher dry strength than the oak samples. Compared to their corresponding dry strength, water soaking resulted in about a 2/3 decrease in the bonding strength of WCSM products with both oak and fir specimens. Re-drying of the wet oak and fir pairs doubled the shear strength of the wet samples. In other words, 1/3 of the lost strength of WCSM due to soaking was able to be recovered after those samples were re-dried. These observations were similar to the trend of laboratory-prepared WCSM samples observed with maple wood samples, although the degree of the lost strength was lower with the latter [35].

The relative bonding strengths of the three WCSM samples were in the same order for all four test conditions: 0.5-mm hammer ≥ 0.18-mm bead > 0.5-mm cyclone. However, no statistically significant (*p* > 0.05) difference was observed between the three values of dry strength. The particle size seemed to have a greater impact on the post-bonding treatments with heat or water. This result was probably due to the fact that protein, the predominant component in WCSM, played a major role as the adhesive agent. Similarly, the more rigid and insoluble carbohydrate components played minor roles as fillers and cross-linkers. Previously, it has been reported that sorghum lignin and lignin-based resin improved the wet strength of soy protein adhesives due to the increased wetting potential of adhesive to wood surfaces, and cross-linking between lignin-based resin and reactive groups on soy protein [4,36]. Pradyawong et al. [37] demonstrated the improvements in bonding performance and the physicochemical properties of soy protein adhesives by added lignin with large (35.66 μm), medium (19.13 μm), and small (10.26 μm) particle sizes. They observed that lignin with smaller particle size increased the wet shear strength of soy protein adhesives, and attributed it to a larger surface area of lignin was available to interact with the protein. Whereas our observations also showed that the particle size of the WCSM adhesives impacted the wet (as well as heat) strength, the WCSM adhesives with fine particles indeed lowered the wet and soaked strength. This difference in observation could be caused by the different size ranges we tested than Pradyawong et al. [32], and/or other non-lignin carbohydrates in WCSM, as reported by Premjai et al. [38]. The earlier work [33] reported that cellulose-rich bagasse residue could effectively be used as reinforcement in bio-based composite materials with wheat gluten. The flexural strength and strain at break of the composites tended to decrease when the size of the bagasse particles used was increased in their three tested particle fractions (2.5–0.5 mm, 0.50–0.25 mm, and <0.25 mm). However, the modulus of elasticity was likely to increase with the larger bagasse particle sizes. In addition, we observed the overheat phenomena with the additional grinding with the laboratory cyclone and ball mills, which might have caused heat denaturation of some proteins, leading to the decreased wet and hot bonding strength of the latter two WCSM samples. The overheat concern may not be so serious in a larger-scale grinding process.

### 3.5. Microscopic Examination of the Bondline Features

**Figure 6:**
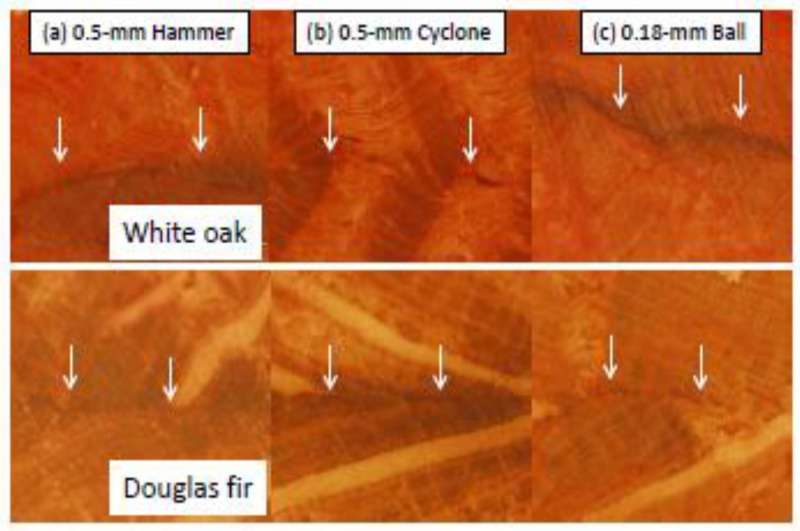
Stereomicroscopic images of white oak and Douglas fir substrates bonded by WCSM products ground by three mills and passed by 0.5-mm and 0.18-mm screens, respectively. For clarity, the horizontal bond line in each image is indicated with two arrows.

Microscopic imaging was used to examine the bonding line thickness and adhesive penetration [39,40]. The images of the cross-sections of oak and fir substrates bonded by WCSM adhesives are presented in Figure 6. The bonding lines were visible along the dark red-colored curves indicated by the arrows. They were generally quite solid and thin, indicating the fair interfacial contact of the adhesives on the two substrates. However, it was hard to clearly identify the adhesive penetration, due to the strong background red color of the lignin in the wood substrate [41]. Although an uneven bondline along the interface of the substrates suggested adhesive penetration occurred, no substantial differences in either bondline features or adhesive penetration were observed between the same type of wood pairs bonded with the WCSM products from the three mills. On the other hand, the bondlines of the Douglas fir substrates looked thinner than those of the white oak specimens, suggesting a more solid contact of the WCSM adhesives with the fir substrates. This observation was consistent with the higher shear strength of the fir strips when compared to that of the oak strips (Figure 4 and Figure 5). Overall, the bonding strength in the two types of wood were comparable to that of petro-chemical glue.

### 3.6. Speculation for Practical Applications

This research provided information on the impacts of the grinding treatments on the physicochemical and adhesive properties of WCSM, which would be helpful in the set-up of industrial production of WCSM for practical bonding applications. The present WCSM-only adhesive could be used for non-structural interior applications. For a wider application, its water resistance should be further improved. This could be done by blending WCSM with wet strength additives, such as tung oil [40], or synthetic epoxy resins [11,42], although more research is needed. In addition, it should be aware that the press time for wood bonding is variable in industrial application. In this work, a 20-min press time was used, as per our previous studies [16,43]. For general plywood and composite board bonding, a shorter fixed press time (3.6–6.0 min) has been used in the literature [16,42,44]. On the other hand, variable press times have also been used to evaluate the adhesives per European Union standards, such as 5, 15, 25 min in Nordqvist et al. [45], and 30 min in He and Chiozza [21]. As a matter of fact, per our industrial partner’s input, 60-, 120- and 180-min press times have been applied in the evaluation of WCSM as a glue for bonding small wood items, such as the pencil sandwich slats in pencil making [19,46]. 

## 4. Conclusions

There are two types of particles in WCSM products. Additional grinding produced more fine particles, altered the morphology of these particles with more fracturing and overlapping features, and enriched the mineral contents in the particle surface.

Data of the dry, hot, wet, and soaked adhesive strengths demonstrated that pilot-produced WCSM products could be suitable for plywood wood bonding with non-structural internal applications. However, more rigorous experimental data are needed to make the conclusion solid. The dry adhesive strength was not affected by the grinding methods in the WCSM preparation. However, additional grinding to smaller homogenous fine particles by cyclone and ball mills did indeed decrease the hot and wet strengths of WCSM adhesives. The decrease could be due to the overheating during the additional grinding which led to some protein being denatured. 

Future work will be test the effect of the particle size on the dispersal potential and dispensability of WCSM products in spray-based applications, such as its utility in particleboard bonding.

## Figures and Tables

**Table 1:** Elemental composition (% atom ± δ) of the cuboid- and filament-like particles of WCSM products by SEM-EDS analysis.

	0.5-mm Hammer		0.5-mm Cyclone		0.18-mm Ball	
Element	Chunk	Filament	Chunk	Filament	Chunk	Filament
C	65.4 ± 0.4	70.5 ± 0.2	63.2 ± 0.3	65.0 ± 0.3	65.0 ± 0.2	68.1 ± 0.2
O	32.5 ± 0.4	28.5 ± 0.2	33.9 ± 0.3	34.6 ± 0.3	30.5 ± 0.2	31.4 ± 0.2
K	1.5 ± 0.0	0.3 ± 0.0	1.5 ± 0.0	0.2 ± 0.0	1.2 ± 0.0	0.2 ± 0.0
Mg	0.2 ± 0.0	0.2 ± 0.0	0.5 ± 0.0	0.1 ± 0.0	1.0 ± 0.0	0.1 ± 0.0
Ca	0.0 ± 0.0	0.1 ± 0.0	0.3 ± 0.0	0.3 ± 0.0	0.2 ± 0.0	0.0 ± 0.0
S	0.0 ± 0.0	0.0 ± 0.0	0.1 ± 0.0	0.0 ± 0.0	0.3 ± 0.0	0.0 ± 0.0
P	0.1 ± 0.0	0.1 ± 0.0	0.1 ± 0.0	0.0 ± 0.0	1.5 ± 0.0	0.0 ± 0.0
Cu	0.2 ± 0.0	0.2 ± 0.0	0.2 ± 0.0	0.1 ± 0.0	0.2 ± 0.0	0.1 ± 0.0
